# Co-expression of GAD67 and choline acetyltransferase reveals a novel neuronal phenotype in the mouse medulla oblongata

**DOI:** 10.1016/j.autneu.2015.05.003

**Published:** 2015-12

**Authors:** Jittima Gotts, Lucy Atkinson, Ian J. Edwards, Yuchio Yanagawa, Susan A. Deuchars, Jim Deuchars

**Affiliations:** a School of Biomedical Sciences, Faculty of Biological Sciences, University of Leeds, Leeds, LS2 9JT United Kingdom; b Department of Genetic and Behavioural Neuroscience, Gunma University Graduate School of Medicine, 3-39-22 Showa-machi, Maebashi 371-8511, Japan

**Keywords:** NTS, Autonomic, Reticular formation, GAD67, ChAT, Brainstem

## Abstract

GABAergic and cholinergic systems play an important part in autonomic pathways. To determine the distribution of the enzymes responsible for the production of GABA and acetylcholine in areas involved in autonomic control in the mouse brainstem, we used a transgenic mouse expressing green fluorescent protein (GFP) in glutamate decarboxylase 67 (GAD67) neurones, combined with choline acetyl transferase (ChAT) immunohistochemistry. ChAT-immunoreactive (IR) and GAD67-GFP containing neurones were observed throughout the brainstem. A small number of cells contained both ChAT-IR and GAD67-GFP. Such double labelled cells were observed in the NTS (predominantly in the intermediate and central subnuclei), the area postrema, reticular formation and lateral paragigantocellular nucleus. All ChAT-IR neurones in the area postrema contained GAD67-GFP. Double labelled neurones were not observed in the dorsal vagal motor nucleus, nucleus ambiguus or hypoglossal nucleus. Double labelled ChAT-IR/GAD67-GFP cells in the NTS did not contain neuronal nitric oxide synthase (nNOS) immunoreactivity, whereas those in the reticular formation and lateral paragigantocellular nucleus did. The function of these small populations of double labelled cells is currently unknown, however their location suggests a potential role in integrating signals involved in oromotor behaviours.

## Highlights

• A small number of neurones in the medulla oblongata contained the synthesising enzymes for both acetylcholine and GABA
• Double labelled cells were found in the NTS, area postrema, reticular formation and lateral paragigantocellular nucleus
• Dual labelled neurones in the reticular formation and lateral paragigantocellular nucleus could also express nNOS

## 1 Introduction

The dorsomedial medulla oblongata contains the nucleus tractus solitarius (NTS) and the area postrema (AP), areas which are both important in central control of a wide range of homeostatic reflexes (Loewy, 1990). Determining the constituent cell types in these areas is therefore important for a full understanding of the circuits involved in central autonomic control. Two of the major neurotransmitters in the NTS and AP are ɣ-amino butyric acid (GABA) (Fong et al., 2005, Walberg and Ottersen, 1992) and acetylcholine (ACh) (Ruggiero et al., 1990, Tago et al., 1989), but their co-localisation has not been reported.

GABAergic systems play an important part in autonomic pathways and blockade of GABAergic signalling in the NTS broadly impacts cardiovascular (Mei et al., 2003), respiratory (Wasserman et al., 2002) and gastrointestinal regulation (Travagli et al., 2006). Immunohistochemistry for GABA or its synthesising enzyme glutamate decarboxylase (GAD) 67 reveals GABAergic neurones and terminals throughout the NTS (Fong et al., 2005, Izzo et al., 1992, Kawai and Senba, 1999, Maqbool et al., 1991, Okada et al., 2008). Similarly in transgenic animals in which enhanced green fluorescent protein expression was linked to GAD67 expression, labelled neurones were widespread across the NTS, and the majority were directly activated by solitary tract afferents (Bailey et al., 2008).

A functional cholinergic system has also been described in the NTS. Microinjection of ACh into the rat NTS induces dose-dependent baroreflex-like hypotensive and bradycardic responses (Criscione et al., 1983, Da Silva et al., 2008, Tsukamoto et al., 1994), modulates sympathetic and phrenic nerve activity (Furuya et al., 2014) and elicits a swallowing response (Hashim and Bieger, 1987).

Anatomically, some NTS neurones and terminals have been shown to contain the ACh hydrolysing enzyme – acetylcholinesterase (Kobayashi et al., 1978) and the enzyme responsible for synthesising Ach – choline acetyl transferase (ChAT) (Armstrong et al., 1988, Helke et al., 1983, Ruggiero et al., 1990, Simon et al., 1985). Two populations of ChAT immunoreactive (IR) neurones in the NTS have been described, one in medial NTS just dorsal to the DVN and one surrounding the medial and dorsal borders of the tractus (Armstrong et al., 1988).

The area postrema is situated on the dorsal part of the medulla oblongata in close proximity to the fourth ventricle. It is classified as a circumventricular organ since the endothelial cells in the capillaries of the area postrema are fenestrated, enabling substances such as proteins to cross the blood brain barrier (Van Der Kooy and Koda, 1983, Wuchert et al., 2008). This region is a significant component in circuits involved in autonomic regulation, for example, it receives afferent inputs from the hypothalamic paraventricular nucleus (PVN), lateral parabrachial nucleus (PBN), NTS (Shapiro and Miselis, 1985) and vagus nerve (Contreras et al., 1982) and projects to the PBN, the dorsal motor nucleus of the vagus, the NTS (Shapiro and Miselis, 1985, Van Der Kooy and Koda, 1983) and the nucleus ambiguus (Shapiro and Miselis, 1985).

Although GABA-IR (Walberg and Ottersen, 1992) and ChAT-IR (Tago et al., 1989) neurones have been observed in the rat NTS and area postrema, to date there has been no report on the co-localisation of the enzymes responsible for synthesising for both ACh and GABA within neurones in this region. A potential reason for this is the difficulty in labelling GABAergic neurones in the brainstem with immunohistochemistry. We therefore utilised a transgenic mouse in which GAD67 is reported by expression of GFP and combined this with immunohistochemistry for ChAT to identify dual labelled neurones in the NTS, area postrema, medullary reticular formation and lateral paragigantocellular nucleus.

## 2 Methods

Adult GAD67-GFP knock-in mice of either sex (4–6 weeks) expressing GFP under control of the endogenous promoter for GAD67 (Tamamaki et al., 2003) were used in line with the Animals (Scientific Procedures) Act 1986 and the ethical standards set out by the University of Leeds Ethical Review Committee by individuals with UK Home Office approval. Every effort was made to minimise the number of animals used and their suffering.

### 2.1 Immunohistochemistry

GAD67-GFP knock-in mice (n = 4) were anaesthetised with sodium pentobarbitone (60 mg/kg) I.P. and perfused transcardially with 4% paraformaldehyde (PFA). Brainstems were dissected and post-fixed in 4% PFA overnight before washing in 0.1 M phosphate buffer (PB). The brainstems were cut at 30–50 μm using a vibrating microtome (Leica VT1300S).

To enhance GFP visualisation sections were incubated in anti-GFP antibody (1:1000 in PBS + 0.1% Triton, rabbit, Invitrogen) overnight at 4 °C, washed 3 × 10 min in PBS and then incubated in AlexaFluor^488^ donkey anti-rabbit (1:1000, Invitrogen) for 1–3 h at room temperature before washing for 3 × 10 min in phosphate buffered saline (PBS). To identify neurones containing ChAT-IR, sections were then incubated in anti-ChAT antibody (1:500 goat, Millipore in PBS + 0.1% Triton) overnight at 4 °C, washed 3 × 10 min in PBS and then incubated in AlexaFluor^555^ donkey anti goat (1:1000, Invitrogen) for 1–3 h at room temperature. Sections were then washed 3 × 10 min in PBS before mounting onto slides and covered with coverslips using Vectamount medium (Vector labs).

To identify neuronal nitric oxide synthase (nNOS) containing neurones, sections were incubated in nNOS antibody (1:250 in PBS, raised in mouse, Santa Cruz) overnight at 4 °C, washed 3 × 10 min in PBS followed by incubation in biotinylated horse anti mouse (1:500, Jackson Immunoresearch) overnight at room temperature. Sections were then washed for 3 × 10 min in PBS and then incubated in Streptavidin Pacific Blue (1:1000, Invitrogen) for 1 h at room temperature. Sections were then washed 3 × 10 min in PBS before being placed onto slides and mounted with coverslips as above.

### 2.2 Microscopy and image capture

Sections were observed under a Nikon Eclipse E600 microscope equipped with epifluoresence and images captured using a Q-Imaging Micropublishing 5.0 camera and Aquis image capture software. Some sections were observed under a confocal microscope (Zeiss LSM 510 Meta) and images captured using Zen software. Images were adjusted for brightness and contrast using CorelDraw x6 software.

### 2.3 Quantification of co-localisation

ChAT-IR and GAD67-GFP containing cells in the NTS were manually quantified from 3 mice. From each animal, every sixth consecutive serial section (50 μm thick) was studied to avoid the risk of double counting the same neurones in adjacent sections. Six sections were counted in total (from each animal); two each representative of the caudal, intermediate and rostral regions of the NTS, and if a section to be investigated was damaged, it was substituted in the analysis by an adjacent section. Due to the limited distribution of the central subnucleus in the NTS, ChAT-IR, GAD67-GFP and nNOS-IR containing cells in this area were counted in four sections, from every third consecutive serial section (30 μm thick). Double labelled cells were determined per section (Table 1) and pooled across all animals. The percentage colocalisation was calculated from pooled data.

## 3 Results

### 3.1 ChAT-IR and GAD67-GFP neurones are present in the NTS and a small number are double labelled

**Fig. 1:**
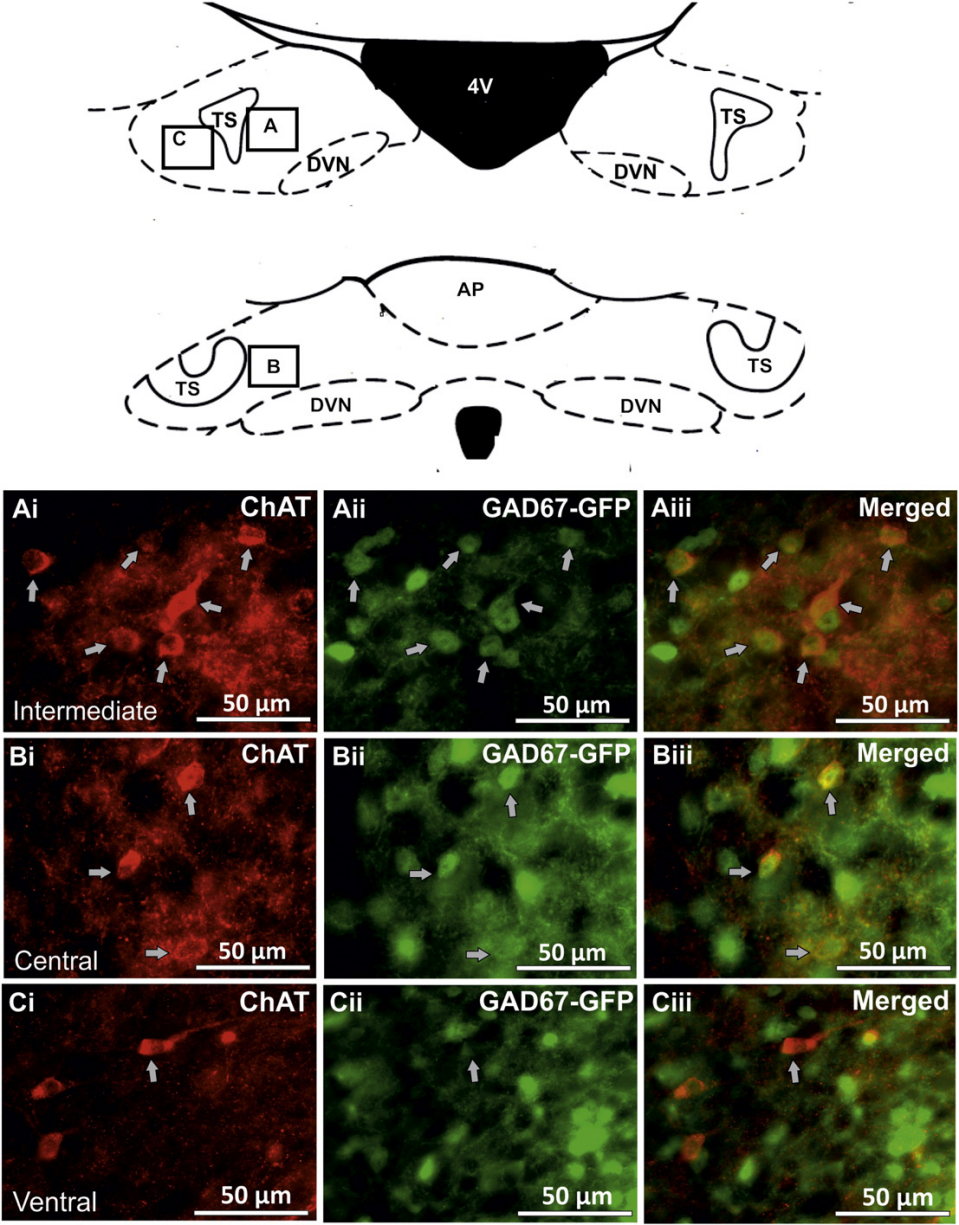
Distribution of ChAT-IR and GAD67-GFP cells in rostral and intermediate NTS. Fluorescent images showing the distribution of ChAT-IR and GAD67-GFP cells in the intermediate (Ai–Aiii), central (Bi–Biii) and ventral (Ci–Ciii) subnuclei of the rostral and intermediate NTS. In all of these NTS subnuclei, double labelled cells containing both ChAT-IR (Ai, Bi and Ci) and GAD67-GFP (Aii, Bii and Cii) can be observed (Aiii, Biii and Ciii). Arrows indicate double labelled neurones. The schematic diagram is adapted from Paxinos and Watson (Paxinos and Watson, 1997) and indicates the approximate position of the subnuclei.

ChAT-IR neurones could be found throughout the subnuclei of the NTS. The cell bodies of ChAT-IR cells were oval or elliptical with a mean size of 12.33 ± 0.49 × 15.68 ± 0.8 μm (n = 16 neurones randomly chosen from all NTS subnuclei). GAD67-GFP containing neurones were also found throughout the NTS with oval or elliptical shaped cell bodies and with a mean size of 10.03 ± 0.31 × 12.59 ± 0.49 μm (n = 12 neurones randomly chosen from all NTS subnuclei). Fig. 1 shows examples of GAD67-GFP and ChAT-IR cells in various NTS subnuclei at rostral and intermediate levels.

A limited number of NTS neurones contained both ChAT-IR and GAD67-GFP. These neurones had small cell bodies (8.15 ± 0.48 × 12.57 ± 0.67 μm, n = 12). The highest percentage of double labelled cells was found in the intermediate subnucleus (81 co-localised/610 total number of ChAT or GAD cells, 13.28%, n = 6 sections, N = 3 animals, Fig. 1 Ai–Aiii), followed by the central subnucleus (19/215, 8.84%, n = 6, N = 3, Fig. 1 Bi–Biii), the ventrolateral subnucleus (10/330, 3.03%, n = 12, N = 3), the ventral (10/343, 2.92%, n = 12, N = 3, Fig. 1 Ci–Ciii), the dorsomedial (13/556, 2.34%, n = 11, N = 3), the dorsolateral (3/247, 1.21%, n = 12, N = 3), the medial (6/924, 0.65%, n = 18, N = 3) and the commissural (2/479, 0.42%, n = 12, N = 3) subnuclei. No double labelled cells were observed in the lateral (0/141, 0%, n = 12, N = 3) or interstitial subnuclei (0/31, 0%, n = 6, N = 3). See Table 1 for the average number of cells observed per 50 μm section in each subnucleus of the NTS.

**Fig. 2:**
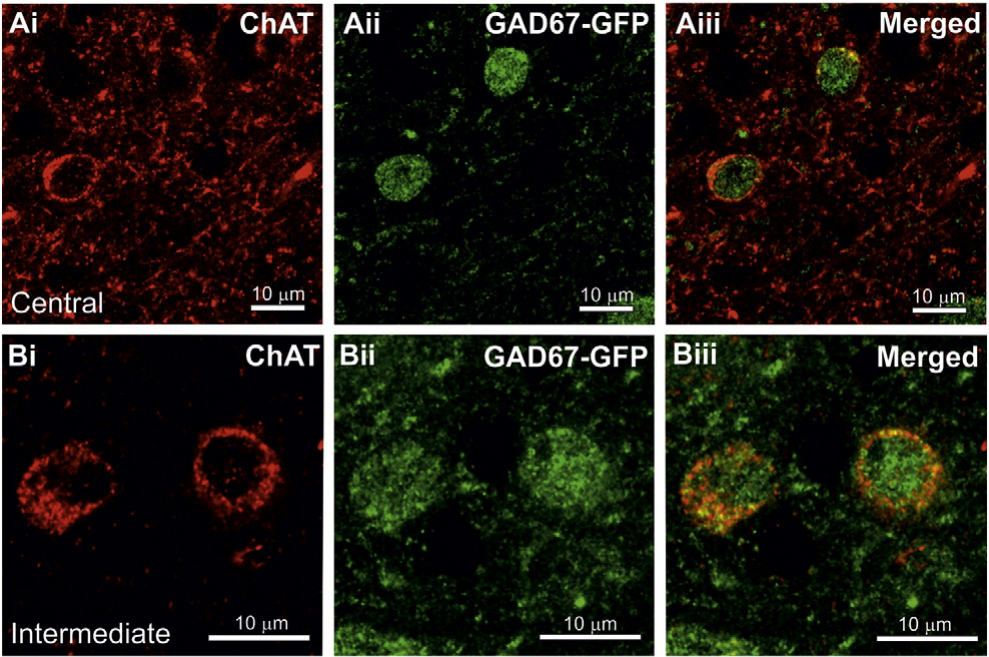
Examples of ChAT-IR and GAD67-GFP double labelled cells in the central and intermediate subnuclei of the NTS. Confocal images showing ChAT-IR is localised to the cytoplasm of the cells in the central (Ai) and intermediate (Bi) subnuclei of the NTS whereas GAD67-GFP is present in the nucleus and the cytoplasm (Aii, Bii).

When examined under a confocal microscope, GAD67-GFP staining was observed throughout the cytoplasm and nucleus of the cells whereas ChAT staining was observed in the cytoplasm (Fig. 2 A-B).

### 3.2 ChAT-IR/GAD67-GFP containing cells in the central subnucleus of the NTS do not contain nNOS

**Fig. 3:**
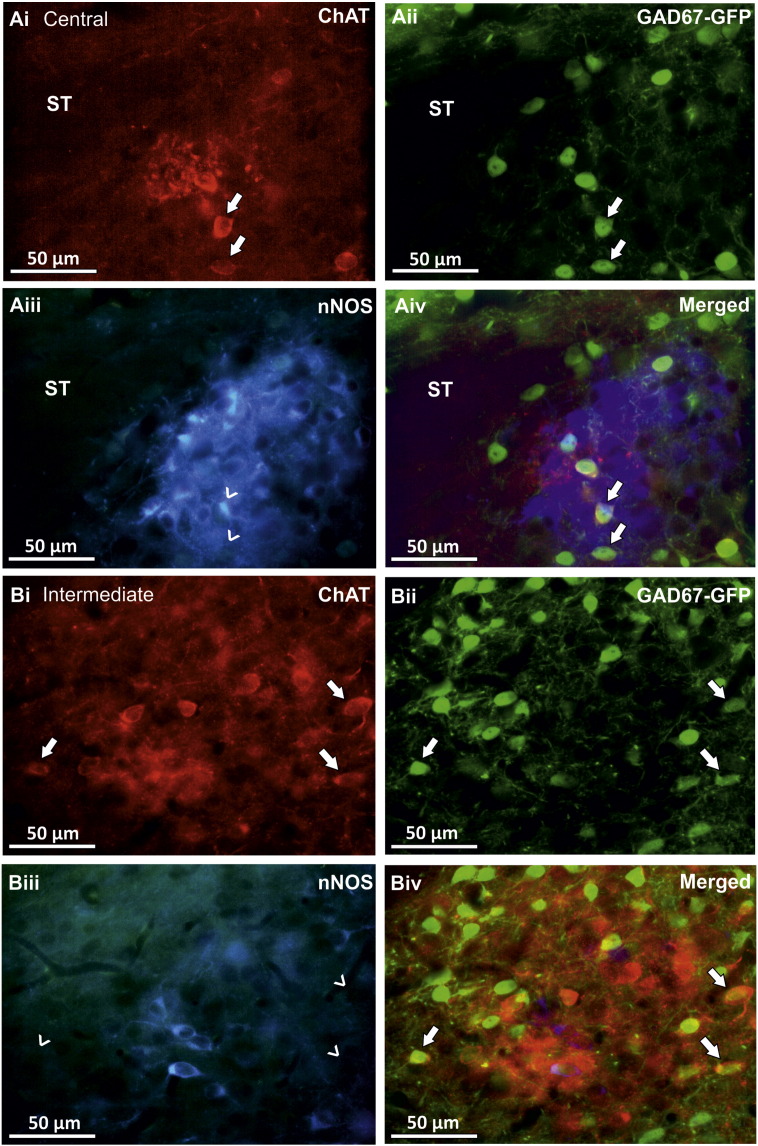
ChAT-IR/GAD67-GFP positive cells in the NTS do not contain nNOS immunoreactivity. Fluorescent images illustrating that cells in the central (Ai–Aiv) and intermediate (Bi–Biv) subnuclei of the NTS contain both ChAT-IR and GAD67-GFP (arrows), but do not contain nNOS-IR (open arrows).

Nitric oxide is the neurotransmitter of the majority of premotor neurones in the central NTS (Wiedner et al., 1995). However, although ChAT-IR and GAD67-GFP double labelled cells were in close proximity to nNOS-IR neurones, they were not observed to contain nNOS immunoreactivity (Fig. 3 Ai–Aiii) in the central subnucleus or any other subnuclei in the NTS (Fig. 3 Bi–Biii).

### 3.3 Area postrema

**Fig. 4:**
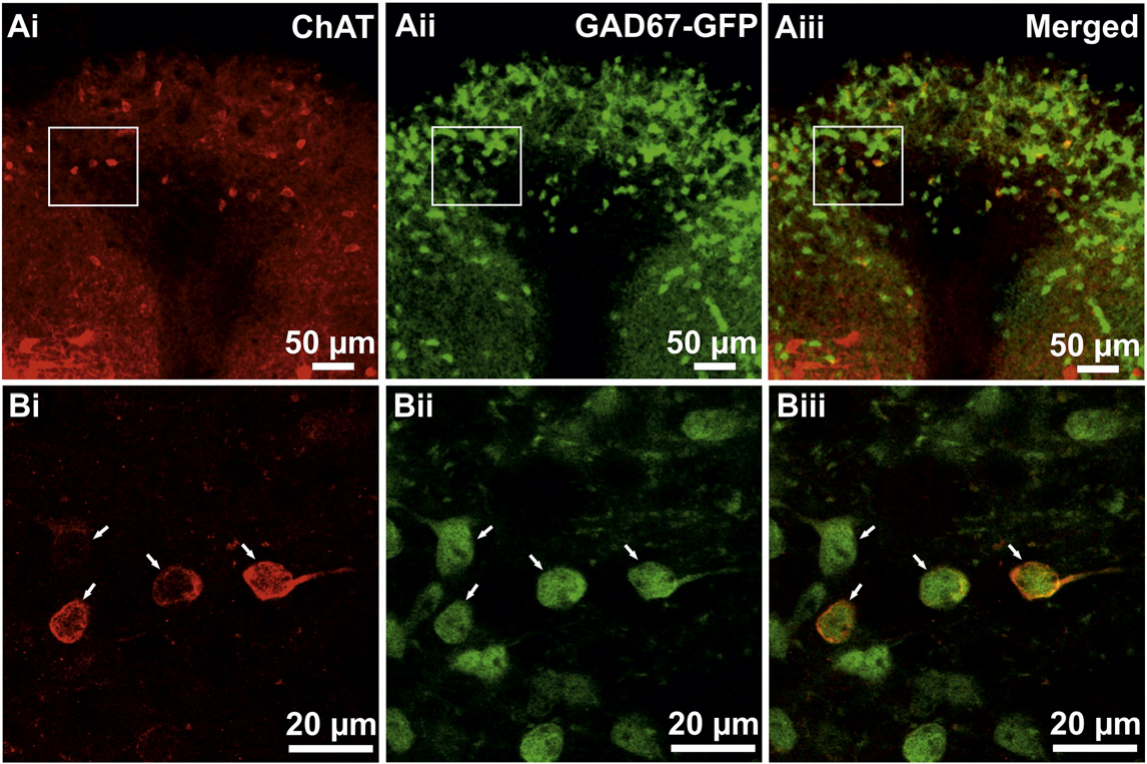
All ChAT-IR neurones in the area postrema contain GAD67-GFP. Confocal images showing ChAT-IR and GAD67-GFP containing cells are found in the area postrema (Ai–Aii). Magnified views of the marked area in A are shown in B illustrates cells that contain both ChAT-IR (Ai, Bi) and GAD67-GFP (Aii, Bii) (Arrows indicate double labelled neurones).

In the area postrema, both ChAT-IR and GAD67-GFP containing neurones were observed (Fig. 4 A, B). The ChAT-IR and GAD67-GFP positive neurones in the region both had oval or elliptical shaped cell bodies, the sizes of which were 9.35 ± 0.43 × 12.77 ± 0.96 μm (n = 5) for ChAT-IR neurones and 9.26 ± 0.24 × 13.49 ± 0.60 μm (n = 5) for GAD67-GFP containing neurones.

A number of neurones in the area postrema contained both ChAT-IR and GAD67-GFP (Fig. 4 Ai–Aiii, Bi–Biii). The size of these neurones was 10.5 ± 0.49 x 14.2 ± 0.97 (n = 5). The mean number of co-localised neurones per 50 μm section was 8.83 ± 5.12 (n = 6 sections, N = 3 animals). All ChAT-IR neurones also contained GAD67-GFP. The percentage of double labelled neurones in all ChAT-IR or GAD-67-GFP neurones in the area postrema is 4.55% (53/1164 cells, n = 6, N = 3).

### 3.4 Reticular formation and its adjacent regions

**Fig. 5:**
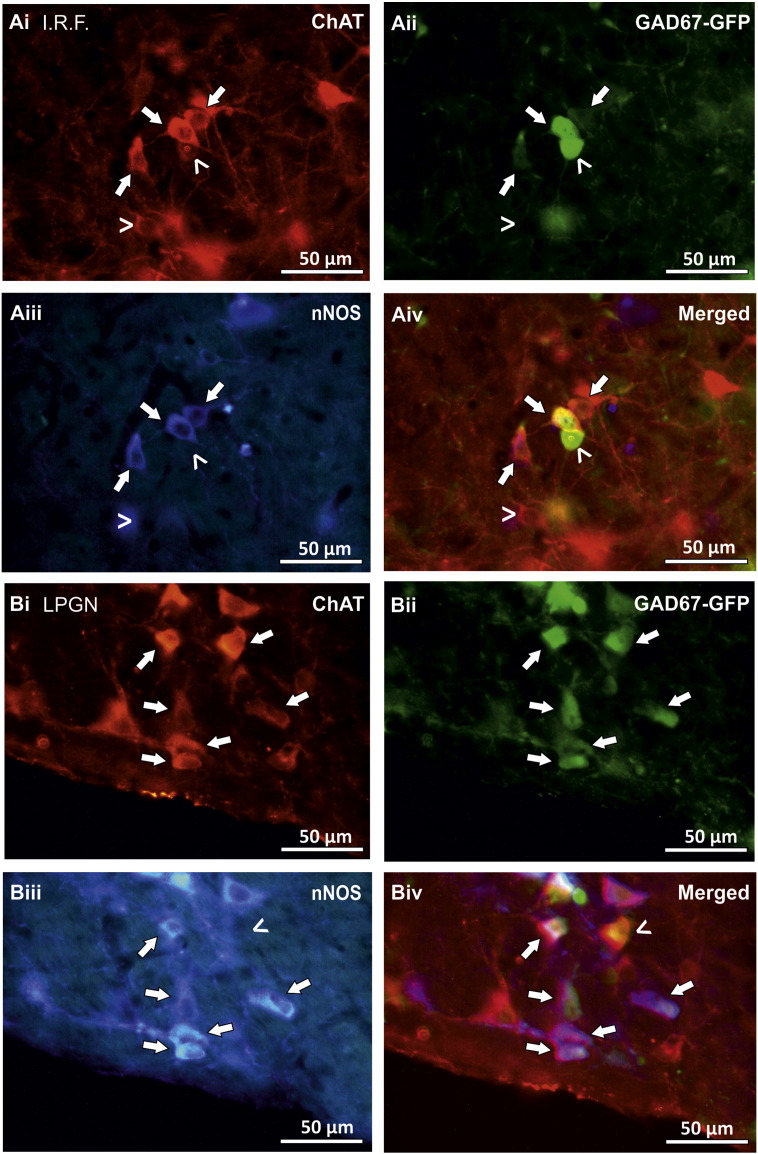
Double labelled GAD67-GFP/ChAT-IR cells are observed in the intermediate reticular formation and the lateral paragigantocellular nucleus, some of which contain nNOS immunoreactivity. Fluorescent images showing that neurones in intermediate reticular formation (I.R.F) (A) and the lateral paragigantocellular nucleus (B) contain both ChAT-IR (Ai, Bi) and GAD67-GFP (Aii, Bii) (arrows). Some of these co-localised neurones also contain nNOS-IR (Aiii, Biii). Open arrows in A show neurones that are not double and/or triple labelled. Open arrows in B show a neurone that is not triple labelled.

Neurones which contained both ChAT-IR and GAD67-GFP could also be found throughout the reticular formation. The size of these neurones was 12.06 ± 0.41 × 15.03 ± 0.73 μm (n = 4) (Fig. 5 Ai–Aiv). The mean number of double labelled ChAT-IR and GAD67-GFP containing neurones per 50 μm section was 5.56 ± 1.29 and 5 ± 1.10 for the right and left side of the section respectively (n = 18 sections, N = 3 animals), which was approximately 2% (190/> 8903) of the total ChAT-IR and GAD67-GFP-IR neurones. Double labelled ChAT-IR and GAD67-GFP containing neurones were also observed in the lateral paragigantocellular nucleus (Fig. 5 Bi–Bii and Biv). These were 11.09 ± 0.94 × 20.03 ± 1.42 μm in size (n = 5) (Fig. 5 Bi–Biv). In the intermediate reticular formation and the lateral paragigantocellular nucleus occasional double labelled ChAT-IR/GAD67-GFP neurones were also observed to contain nNOS (Fig. 5 A, B).

### 3.5 The dorsal vagal nucleus (DVN)

**Fig. 6:**
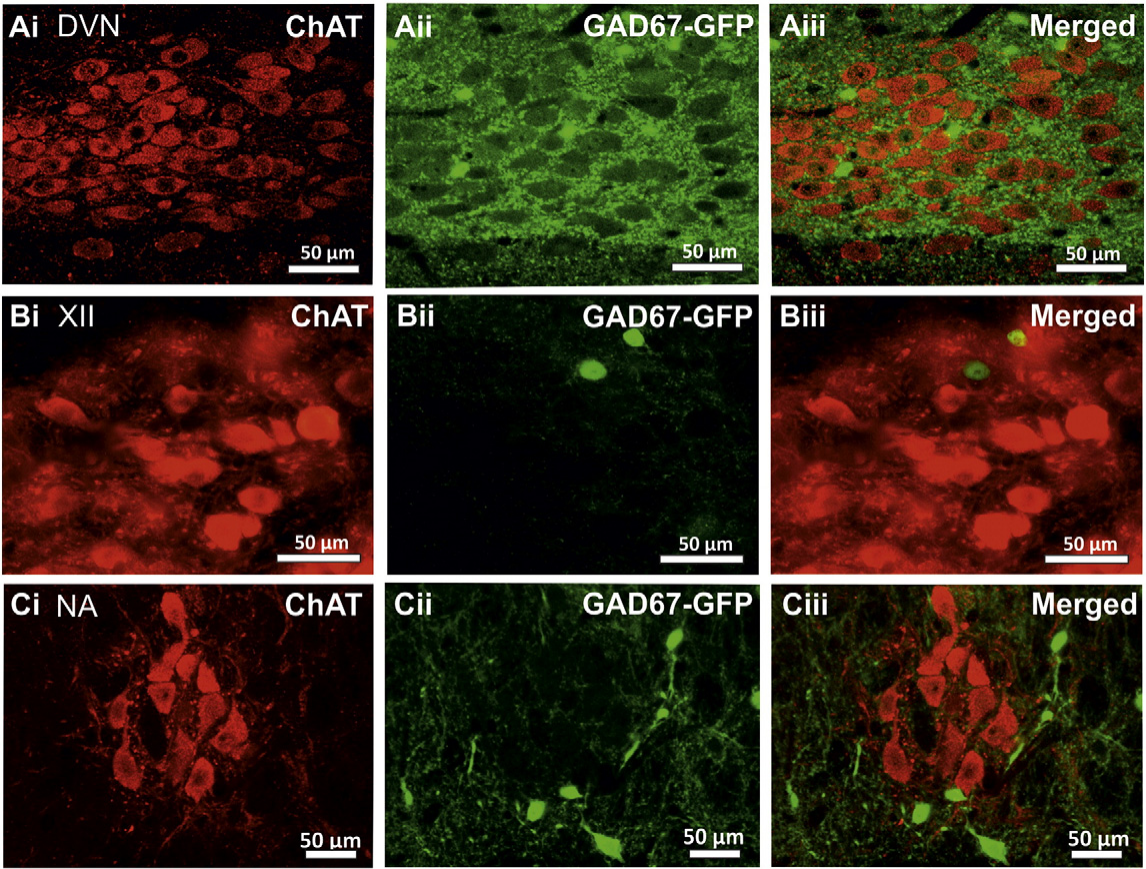
No double labelled GAD67-GFP/ChAT-IR cells in the dorsal vagal nucleus, the hypoglossal nucleus or the nucleus ambiguus. Confocal (A and C) and fluorescent images (B) showing ChAT-IR and GAD67-GFP containing cells are present in the DVN (Ai–Aii), hypoglossal nucleus (Bi–Bii) and the nucleus ambiguus (Ci–Cii) and yet do not co-localise in these areas (Aiii, Biii, Ciii).

In the DVN, a large number of neurones contained ChAT-IR (Fig. 6 Ai). These neurones were large in size (16.45 ± 1.07 × 23.87 ± 0.98 μm, n = 10) and possessed an oval or elliptical shaped cell body, typical of vagal preganglionic neurones. The DVN also contained occasional GAD67-GFP-IR neurones (Fig. 6 Aii), which similarly exhibited oval or elliptical shaped cell bodies, but were smaller in size (10.08 ± 0.58 × 13.86 ± 0.94 μm, n = 10). No cells were observed to contain both GAD67-GFP and ChAT in the DVN.

### 3.6 The hypoglossal nucleus

Many neurones in the hypoglossal nucleus were intensely ChAT-IR. These neurones were large (18.94 ± 0.94 × 25.42 ± 0.83 μm, n = 10) (Fig. 6 Bi). The hypoglossal nucleus also contained a limited number of GAD67-GFP-IR neurones (Fig. 6 Bii). Co-localisation for ChAT and GAD67-GFP was not observed in the hypoglossal nucleus.

### 3.7 Nucleus ambiguus

The nucleus ambiguus contained intensely ChAT-IR neurones whose sizes, at 17.94 ± 0.98 × 20.4 ± 1.25 μm (n = 5), were relatively large. Smaller GAD67-GFP-IR neurones (9.66 ± 0.64 × 13.06 ± 0.89 μm, n = 5) were also found in this area. Co-localisation of ChAT and GAD67-GFP was not observed (Fig. 6 Ci–Ciii).

## 4 Discussion

This study describes subpopulations of neurones containing enzymes responsible for the production of both ACh and GABA in areas of the medulla oblongata involved in autonomic control. Neurones containing both ChAT-IR and GAD67-GFP staining were observed in the NTS, area postrema and the reticular formation of the mouse brainstem. Whilst neurones containing both ChAT and GAD (or GABA) immunoreactivity have previously been observed in neurones in the rat retina, cerebral cortex, basal forebrain and spinal cord (Kosaka et al., 1988), hypoglossal nucleus (Davidoff and Schulze, 1988), turtle retina (Nguyen and Grzywacz, 2000), and the tegmental nuclei of the cat (Jia et al., 2003), these are the first such populations described throughout the medulla oblongata.

### 4.1 ChAT-IR and GAD67-GFP neurones in the NTS

A very small number of neurones containing both ChAT-IR and GAD67-GFP was located throughout the NTS (with the exception of the lateral and interstitial subnuclei). The largest populations of double labelled cells were observed in the intermediate and central subnuclei of the NTS. Their small size may correlate to the small cells with local axonal arborisation and no axonal projections (Kawai and Senba, 1999, Okada et al., 2006) which include GABAergic neurones (Kawai and Senba, 1999) suggesting a potential role in integrating input information in local circuits of the NTS.

Tracing studies have shown that the intermediate subnucleus of the NTS receives afferent input from the tongue (Hayakawa et al., 2001), soft palate, pharynx and larynx (Altschuler et al., 1989, Broussard and Altschuler, 2000) and contains pharygeal premotor neurones. The central subnucleus of the NTS receives the majority of the sensory afferent inputs from the oesophagus (Altschuler et al., 1989) and contains oesophageal premotor neurones that project to the nucleus ambiguus. The small populations of double labelled cells containing both ChAT immunoreactivity and GAD67-GFP in the intermediate and central subnuclei of the NTS may therefore play a role in the integration of several visceral sensory inputs that contribute to the reflex control of swallowing, gagging and phonation.

The vast majority of premotor neurones in the central subnucleus of the NTS that project to the nucleus ambiguus use nitric oxide as a neurotransmitter (Wiedner et al., 1995). The fact that the GAD/ChAT double labelled neurones in the central subnucleus of the NTS do not contain nNOS immunoreactivity may suggest these cells are the small proportion of local interneurones that do not contain NOS. Indeed, it has been suggested that GABA interneurons in the intermediate and central subnuclei of the NTS inhibit the premotor neurons involved in the pharyngeal and esophageal stages of swallowing (Dong et al., 2000, Wang and Bieger, 1991).

It therefore seems likely that in the NTS ChAT/GAD cells are involved in circuitry mediating autonomic reflexes. Future studies may therefore investigate whether these ChAT/GAD cells receive direct innervation from afferents or are otherwise involved in reflex arcs through their outputs.

### 4.2 ChAT-IR and GAD67-GFP neurones in the area postrema

Cells containing both GAD67-GFP and ChAT-IR were observed in the area postrema and they contributed to a small percentage (4.55%, 53/1164) of all the ChAT-IR and GAD67-GFP neurones in this area. The area postrema is a circumventricular organ located outside the blood brain barrier and a vital player in the control of autonomic functions. It receives blood borne information from the vasculature in addition to input from the vagus nerve, carotid sinus and other brain areas and projects to autonomic centres including the NTS, NA, RVLM and DVN (for review see Price et al. (2008)). Previously GABA-IR (Walberg and Ottersen, 1992) and ChAT-IR (Tago et al., 1989) neurones have been observed in the rat area postrema but this is the first study to show a population of neurones which contain enzymes responsible for both Ach and GABA. The area postrema offers an attractive area in which to study the brainstem ChAT/GAD neurones since all ChAT-IR neurones also contained GAD67-GFP. Transgenic mice in which ChAT cells are labelled (Kolisnyk et al., 2013, Krasteva et al., 2012) would therefore enable specific targeting of these cells.

### 4.3 ChAT-IR and GAD67-GFP neurones in the reticular formation

The reticular formation contains neurones involved in a wide range of autonomic and regulatory processes including ingestive responses and a role in organising rhythmic oromotor behaviours (Inoue et al., 1994, St John, 1986, Travers et al., 1997). We observed 2% of neurones containing both ChAT-IR and GAD67-GFP in this area, a subpopulation of which also contained nNOS immunoreactivity. Previously, Travers et al. (Travers et al., 2005) have shown that 6.7% of pre-oromotor neurones in the intermediate reticular formation contained both ChAT and NOS immunoreactivity and suggested that they may represent a subset of GABAergic neurones. This study has shown that there is indeed such a population of triple labelled neurones in the reticular formation, although their function remains to be determined.

### 4.4 Unravelling functions of GAD67-GFP/ChAT neurones

A significant hurdle to unravelling the functions of the dual labelled cells identified here are their small numbers and limited intensity of immunolabelling. However, current transgenic technologies may render these cells amenable to further investigation, for example, the GAD67-GFP mice used in this study could feasibly be crossed with another line of mice bred to express a complementary reporter such as mCherry under control of the ChAT promoter (Nagode et al., 2011, Nelson et al., 2014). It is possible that transgenic technologies may also enable selective expression of optogenetic proteins within these cells thus permitting selective activation/inhibition (Nagode et al., 2011) in future functional investigations. Indeed, recent technology has introduced transgenic mice for intersectional targeting of specific neurone populations for expression of neural sensors and effectors (Madisen et al., 2015), an approach that would be ideal for revealing the functions of dual expressing neurones such as these GAD67-GFP/ChAT-IR cells. Such approaches to reveal functions of these and other cell classes may therefore provide improved understanding of autonomic microcircuitry in the medulla oblongata.

## 5 Conclusion

Small populations of cells in the NTS, area postrema, reticular formation and lateral paragigantocellular nucleus in the mouse medulla oblongata contain the synthesising enzymes for both acetylcholine and GABA. In all areas in the brainstem the function of these cells is currently unknown.

## Figures and Tables

**Table 1:** The number of ChAT and/or GAD67-GFP immunoreactive neurones in each subdivision of the NTS (per 50 μm section) in 3 animals. n = number of sections.

NTS subnucleus	ChAT and GAD67-GFP co-localisation per 50 μm section (mean ± standard error)	ChAT-IR neurones per 50 μm section (mean ± standard error)	GAD67-GFP-IR neurones per 50 μm section (mean ± standard error)
*Intermediate (n = 6)*			
Right	6.8 ± 1.9	17.5 ± 4.3	37.3 ± 8.2
Left	6.7 ± 2.2	16.7 ± 3.3	30.2 ± 6.4

*Central (n = 6)*			
Right	1.0 ± 0.7	2.2 ± 1.3	6.3 ± 2.6
Left	2.2 ± 1.4	2.8 ± 1.6	14.5 ± 2.0

*Ventrolateral (n = 12)*			
Right	0.3 ± 0.1	1.6 ± 0.7	13.3 ± 3.1
Left	0.5 ± 0.3	1.8 ± 0.7	10.8 ± 2.3

*Ventral (n = 12)*			
Right	0.4 ± 0.2	1.1 ± 0.4	13.8 ± 3.3
Left	0.4 ± 0.2	1.0 ± 0.5	12.7 ± 2.3

*Dorsomedial (n = 11)*			
Right	0.6 ± 0.3	4.4 ± 1.7	18.9 ± 3.9
Left	0.6 ± 0.6	4.6 ± 2.1	22.6 ± 5.9

*Dorsolateral (n = 12)*			
Right	0.3 ± 0.2	2.2 ± 1.0	10.2 ± 3.4
Left	0.0 ± 0.0	0.9 ± 0.4	7.3 ± 1.5

*Medial (n = 18)*			
Right	0.2 ± 0.1	2.1 ± 0.6	25.4 ± 4.0
Left	0.2 ± 0.1	1.3 ± 0.3	22.6 ± 3.8

*Commissural*			
(n = 12)	0.2 ± 0.1	5.0 ± 2.0	34.9 ± 9.1

*Lateral (n = 12)*			
Right	0.0 ± 0.0	0.1 ± 0.1	5.9 ± 1.6
Left	0.0 ± 0.0	0.1 ± 0.1	5.7 ± 2.0

*Interstitial (n = 6)*			
Right	0.0 ± 0.0	0.7 ± 0.5	3.3 ± 0.8
Left	0.0 ± 0.0	0.0 ± 0.0	1.2 ± 0.5
